# The 19S proteasome as a therapeutic target in hematologic malignancies

**DOI:** 10.3389/fcell.2026.1918833

**Published:** 2026-10-07

**Authors:** Idaly M. Olivas, Mayra A. Gonzalez-Henry, Saeedeh Darvishi, Evelyn M. Esparza, Anna M. Eiring

**Affiliations:** 1 Department of Biological Sciences, University of Texas at El Paso, El Paso, TX, United States; 2 Paul L. Foster School of Medicine, Texas Tech University Health Sciences Center El Paso, El Paso, TX, United States

**Keywords:** cancer, drug targets, proteasome inhibition, ubiquitin-proteasome system, 19S proteasome, deubiquitinases, hematologic malignancies, multiple myeloma

## Abstract

The ubiquitin–proteasome system (UPS) is the primary intracellular pathway for maintaining protein homeostasis through the selective degradation of ubiquitinated proteins. Central to this process is the 26S proteasome, which orchestrates the turnover of regulatory and misfolded proteins and thereby controls critical cellular functions, including cell-cycle progression, apoptosis, DNA repair, signal transduction, and stress response pathways. Dysregulation of proteasome function contributes to many hallmarks of cancer, leading to malignant transformation, disease progression, and therapeutic resistance, making the proteasome an attractive target for anticancer therapy. Clinically approved proteasome inhibitors, including bortezomib, carfilzomib, and ixazomib, have significantly improved outcomes in multiple myeloma and other hematologic malignancies by targeting the catalytic activity of the 20S core particle. However, their long-term clinical benefit is limited by intrinsic and acquired resistance and treatment-related toxicities. Recent studies have identified the 19S regulatory particle as a mechanistically distinct and therapeutically promising target. Unlike the proteolytic 20S core, the 19S complex controls substrate recognition, deubiquitination, unfolding, and translocation into the proteolytic chamber. Beyond its role in protein degradation, the 19S regulatory particle participates in transcriptional regulation, chromatin remodeling, and DNA damage responses. Several 19S subunits, particularly members of the PSMD and PSMC families, are overexpressed in hematologic and solid malignancies and are associated with poor clinical outcomes. Genetic and pharmacologic targeting of 19S ATPases and deubiquitinating enzymes induces proteotoxic stress, overcomes resistance to conventional proteasome inhibitors, and suppresses tumor growth in preclinical models. In this review, we discuss the biological functions of the 19S regulatory particle, its role in cancer pathogenesis, and emerging therapeutic strategies targeting 19S-associated proteins to improve outcomes in hematologic malignancies.

## 1 Introduction

The 26S proteasome is a large multiprotein complex that functions as the central effector of the ubiquitin-proteasome system (UPS), the primary pathway responsible for regulated intracellular protein degradation and maintenance of proteome homeostasis in eukaryotic cells. The proteasome mediates the turnover of approximately 90% of intracellular proteins and regulates numerous cellular processes, including cell cycle progression, apoptosis, differentiation, and therapeutic responses (Ross et al., 2021). Structurally, the 26S proteasome comprises two major subcomplexes: the 20S catalytic core particle and one or two 19S regulatory particles that cap the ends of the core. These components function coordinately to recognize, unfold, translocate, and degrade ubiquitinated substrates, thereby ensuring protein quality control and regulating numerous cellular processes (Figure 1) (Bard et al., 2018).

**FIGURE 1:**
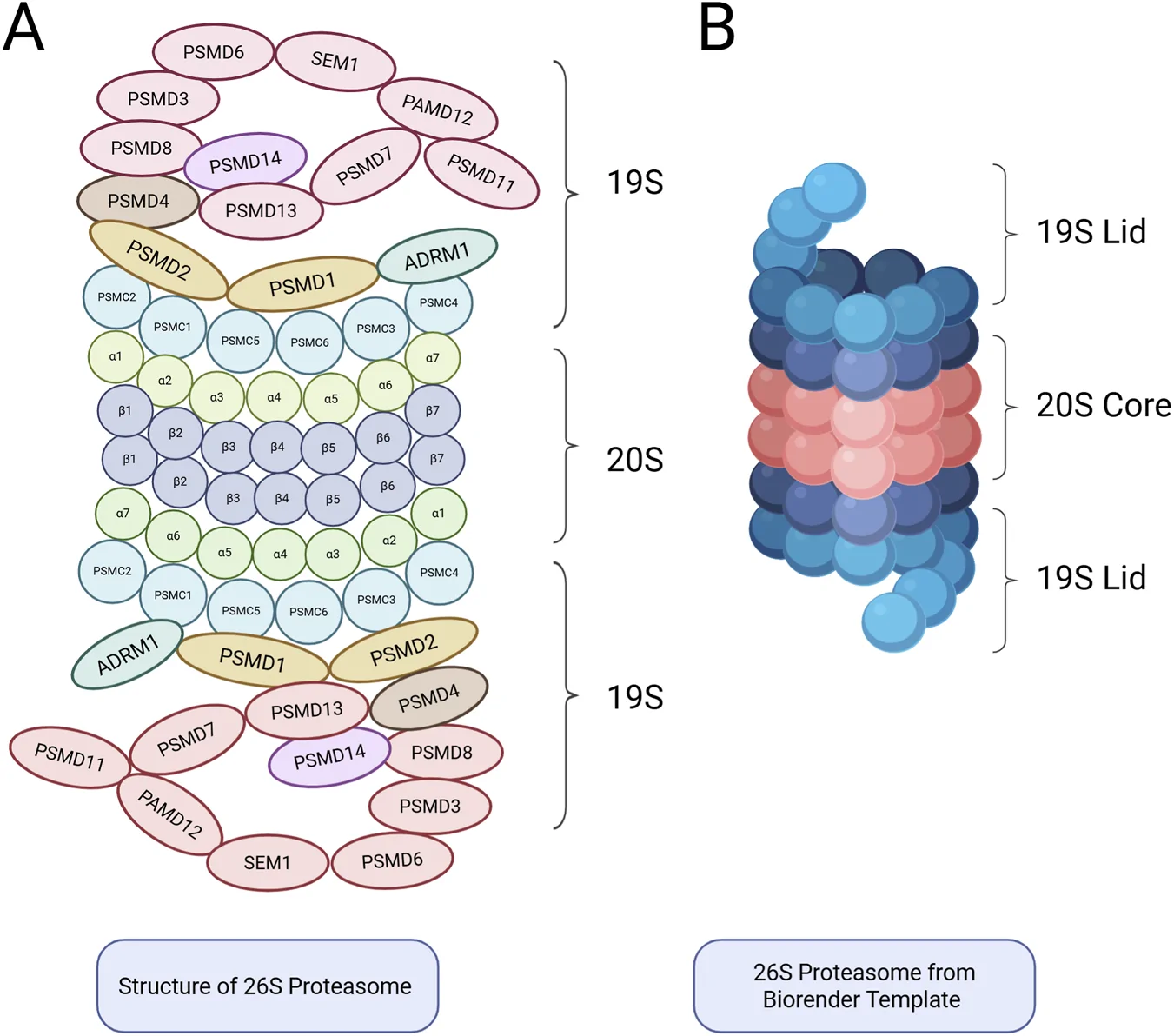
Structure of the 26S proteasome. **(A)** Subunits of the 20S core complex and 19S regulatory complexes are depicted. The 20S core complex consists of α1/PSMA1-α7/PSMA7 and β1/PSMB1-β7/PSMB7. The 19S regulatory complexes contain two structures: a base (Rpt1/PSMC1-Rpt6/PSMC6, Rpn1/PSMD2, Rpn2/PSMD1, Rpn10/PSMD10, and Rpn13/ADRM1) and a lid (Rpn3/PSMD3, Rpn4/PSMD9-Rpn9/PSMD13, Rpn11/PSMD14, Rpn12/PSMD8, and Rpn15/SEM1). Rpn4/PSMD9 acts as a chaperone during assembly of the base of the 19S regulatory complex (Sahu et al., 2014). Redrawn from refs (Lander et al., 2012; Lasker et al., 2012; Stone and Morris, 2014; D'Arcy et al., 2015; Marshall and Vierstra, 2019). **(B)** Figure depicts the 26S proteasome from BioRender. Created in https://BioRender.com.

As the primary effector of the ubiquitin-proteasome system, the proteasome plays a critical role in maintaining protein homeostasis and regulating cellular processes such as cell differentiation, cell-cycle progression, apoptosis, and the development of drug resistance (Bassermann et al., 2014; Rousseau and Bertolotti, 2018; Tychhon et al., 2023). Protein degradation through the UPS occurs via a tightly coordinated cascade beginning with selective substrate recognition, followed by ubiquitination, a post-translational modification that covalently marks proteins for degradation (Ross et al., 2021). Ubiquitin, an 8.6 kDa protein, is conjugated to lysine residues through the sequential actions of E1 (activating), E2 (conjugating), and E3 (ligating) enzymes, while deubiquitinating enzymes (DUBs) dynamically reverse this process to fine-tune substrate turnover (Jeong et al., 2023). Through the coordinated interplay of ubiquitin ligases, DUBs, and the proteasome, damaged, misfolded, and regulatory proteins are efficiently degraded, thereby preserving cellular integrity while controlling signaling networks that drive proliferation, survival, and therapeutic responses in cancer cells.

Proteasomes exhibit substantial structural and functional diversity, forming a spectrum of distinct complexes rather than a single uniform entity. This heterogeneity arises from the incorporation of alternative regulatory particles that are associated with the 20S core complex. Despite this variability, the 20S catalytic core remains the conserved proteolytic engine across all assemblies (Wang et al., 2020). The 20S proteasome is a hollow, barrel-shaped protease composed of four stacked heptameric rings in the α-β-β-α configuration. The two outer α rings regulate substrate entry into the catalytic chamber, whereas the two inner β rings contain the proteolytic active sites (Thomas et al., 2023). Among the β subunits, only three possess proteolytic activity: β1 (PSMB6), β2 (PSMB7), and β5 (PSMB5), which confer caspase-like, trypsin-like, and chymotrypsin-like activities, respectively (Voutsadakis, 2017). The coordinated action of these catalytic sites enables degradation of structurally diverse protein substrates.

Building upon the catalytic function of the 20S core, the 19S regulatory complex, also known as the PA700 complex, associates with one or both ends of the 20S particle to form the functional 26S proteasome (Ross et al., 2021). Assembly occurs through non-covalent interactions, with the 19S particle docking onto the α-rings of the 20S core particle. Although the 19S regulator predominates, alternative caps such as the 11S (PA28) complex can associate under specific conditions, generating hybrid proteasomes that are particularly active during immune and stress responses and contributes to the formation of immunoproteasomes (Figure 2) (Thomas et al., 2023). While proteolysis occurs exclusively within the 20S catalytic chamber, the 19S complex performs essential upstream functions including recognition of polyubiquitinated proteins, deubiquitination, ATP-dependent unfolding, and substrate translocation. Through these coordinated activities, the 19S regulatory particle governs substrate selectivity and processing efficiency, acting as a critical interface between ubiquitination and proteolytic degradation (Table 1).

**FIGURE 2:**
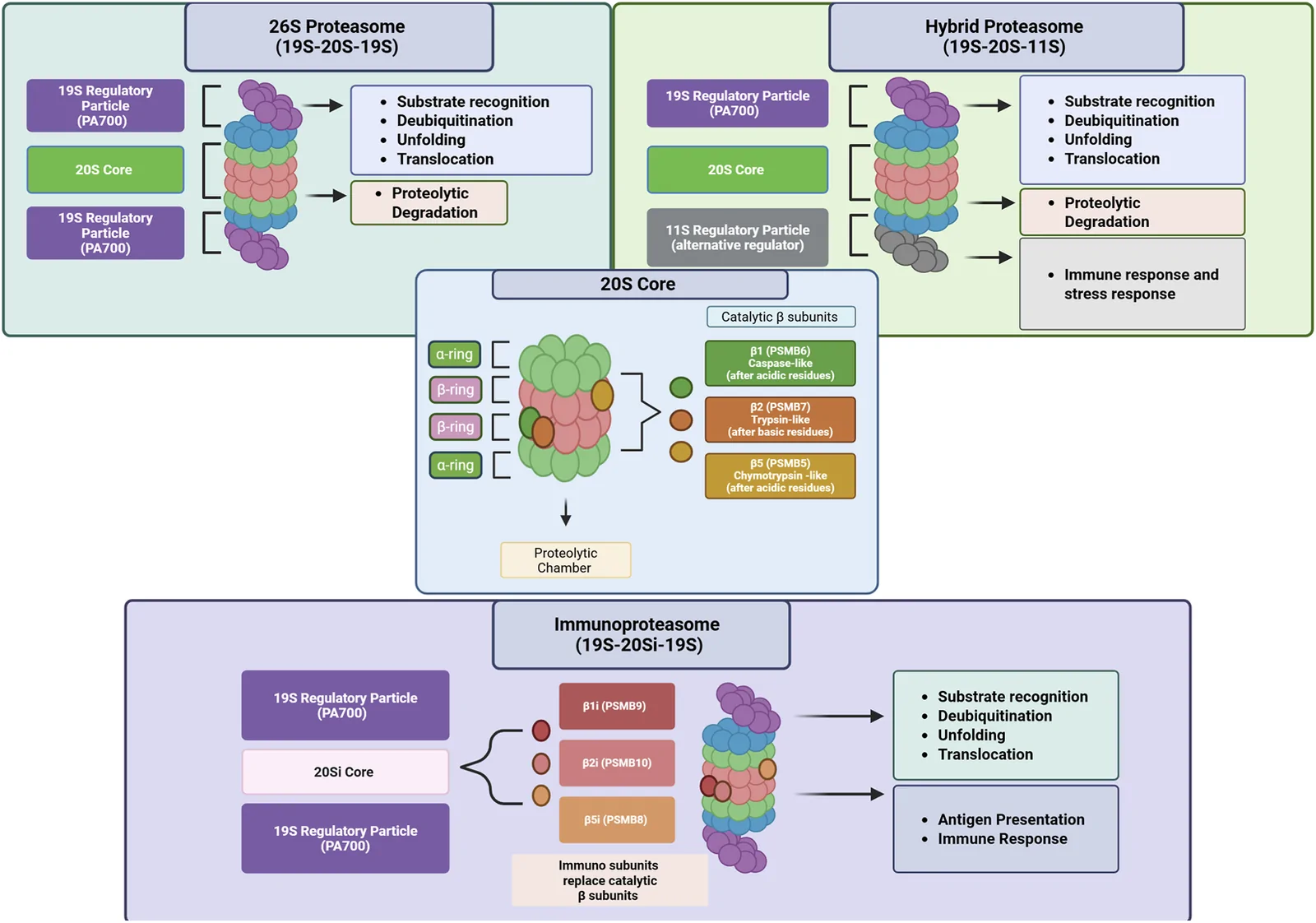
Structural diversity of the 20S proteasome. Structural diversity of proteasomal assemblies, including the standalone 20S core, the canonical 26S proteasome capped by the 19S regulators, hybrid proteasomes containing both 19S and 11S/PA28 activators, and the immunoproteasome, depicting each of their respective functions. Created in https://BioRender.com.

**TABLE 1:** Glossary of terms.

Component	Definition
20S Core	The catalytic component of the proteasome, consisting of four stacked rings (α7β7β7α7) that form a barrel-shaped degradation chamber. The β1, β2, and β5 subunits provide caspase-like, trypsin-like, and chymotrypsin-like activities, enabling the degradation of intracellular proteins and maintenance of proteostasis
19S Base	The 19S base is the ATPase-containing subcomplex of the 19S regulatory particle that docks onto the α-rings of the 20S proteasome. Composed of six AAA + ATPases (Rpt1-Rpt6) and associated non-ATPase subunits, it mediates substrate engagement, ATP-dependent unfolding, gate opening of the 20S core, and translocation of ubiquitinated proteins into the proteolytic chamber for degradation
19S Lid	The 19S lid is the deubiquitination and substrate-processing subcomplex of the 19S regulatory particle. Composed primarily of non-ATPase PSMD subunits, including the metalloprotease PSMD14/Rpn11, it recognizes polyubiquitinated proteins, removes ubiquitin chains, and coordinates substrate transfer to the ATPase-containing 19S base for unfolding and translocation into the 20S proteolytic core
19S ATPases	The 19S ATPases are a hexameric ring of AAA + ATPase subunits (Rpt1-Rpt6; PSMC1-PSMC6) located within the 19S base of the 26S proteasome. Through ATP-dependent conformational changes, these proteins mediate substrate unfolding, gate opening of the 20S core particle, and translocation of ubiquitinated proteins into the proteolytic chamber, thereby enabling efficient proteasomal degradation
19S DUBs	19S deubiquitinating enzymes (DUBs) are proteasome-associated enzymes that regulate the processing of polyubiquitinated substrates by removing or remodeling ubiquitin chains before degradation. Located within the 19S regulatory particle, these enzymes, particularly PSMD14/Rpn11, couple deubiquitination to substrate translocation, promote ubiquitin recycling, and ensure efficient delivery of proteins to the 20S catalytic core
19S Ubiquitin Receptors	19S ubiquitin receptors are substrate-recognition components of the 19S regulatory particle that bind polyubiquitinated proteins and target them to the proteasome for degradation. Receptors such as PSMD4 (Rpn10) and ADRM1 (Rpn13) recognize ubiquitin chains and coordinate substrate delivery to the 19S ATPase machinery, thereby coupling ubiquitin-dependent signaling with proteasomal proteolysis

Given the central role of the UPS in maintaining cellular proteostasis, its dysfunction contributes to the development and progression of numerous diseases, most notably neurodegenerative disorders, infectious diseases, and cancer, making the UPS an attractive target for therapeutic intervention (Edelmann et al., 2011; Narayanan et al., 2020). Cancer cells exhibit an increased reliance on proteasome-mediated protein turnover to sustain rapid proliferation and mitigate proteotoxic stress. This dependency has been therapeutically exploited through the development of proteasome inhibitors, several of which have demonstrated clinical efficacy in hematologic malignancies (Voutsadakis, 2017). However, these agents primarily target the catalytic activity of the 20S proteasome, and their long-term effectiveness is limited by resistance mechanisms and dose-limiting toxicities. These limitations highlight a critical gap in current therapeutic strategies, as inhibition of the 20S catalytic core alone is insufficient to achieve durable suppression of proteasome-dependent oncogenic processes. Increasing evidence suggests that regulatory functions mediated by the 19S particle represent a distinct therapeutic vulnerability. In this review, we propose that targeting the 19S regulatory complex constitutes a mechanistically distinct, resistance-focused therapeutic strategy that extends beyond conventional 20S inhibition, with the potential to overcome established resistance mechanisms and expand proteasome-targeted therapies across cancer types.

## 2 Targeting the UPS in hematologic malignancies

Altered expression of proteasome subunits has been documented across a wide range of hematologic malignancies, including multiple myeloma (MM), diffuse large-B cell lymphoma (DLBCL), mantle cell lymphoma (MCL), and both acute and chronic lymphoblastic leukemia (Di Costanzo et al., 2020; Tubío-Santamaría et al., 2021). In these diseases, proteasome subunits contribute not only to protein quality control, but also to the regulation of critical oncogenic pathways governing cell cycle progression, apoptosis, DNA damage repair, immune signaling, and cellular metabolism. Increased expression of both 20S catalytic and 19S regulatory subunits is frequently associated with enhanced proteolytic capacity, enabling malignant cells to cope with elevated proteotoxic stress resulting from rapid proliferation, high rates of protein synthesis, and genomic instability. In multiple myeloma, heightened dependence on proteasome activity represents a well-established therapeutic vulnerability, reflecting the substantial burden of immunoglobulin production and protein turnover characteristic of malignant plasma cells (Pelon et al., 2024). In acute lymphoblastic leukemia (ALL) and chronic lymphoblastic leukemia (CLL), proteasome dysregulation has been associated with resistance to apoptosis, altered immune responses, and adaptation to cellular stress (Lamothe et al., 2016; Sin and Man, 2021). Likewise, lymphoma cells frequently exploit enhanced proteasome activity to maintain proteostasis under conditions of metabolic stress and oncogene activation (Di Costanzo et al., 2020). Collectively, these observations highlight the multifaceted roles of proteasome subunits in hematologic malignancies and provide a strong rationale for developing therapeutic strategies that target not only the catalytic 20S core but also the regulatory functions of the 19S proteasome.

Clinical experience with proteasome-targeted therapies has revealed both the strengths and limitations of disrupting the UPS in hematologic malignancies. The greatest success has come from 20S catalytic inhibitors, particularly bortezomib, carfilzomib, and ixazomib, which have significantly improved outcomes in multiple myeloma and established proteasome inhibition as a validated therapeutic strategy. However, long-term clinical efficacy is often limited by cumulative toxicities, including peripheral neuropathy, hematologic adverse effects, and gastrointestinal complications, as well as the emergence of acquired resistance. Several lessons have emerged from these clinical experiences. First, cancer cells can adapt to catalytic proteasome inhibition through mutations or overexpression of proteasome subunits, particularly PSMB5, and through activation of compensatory pathways such as autophagy and unfolded protein response signaling. Second, clinical responses are often greatest in malignancies with exceptionally high proteostasis demands, such as multiple myeloma, where dependence on protein degradation creates a therapeutic vulnerability. Third, toxicity from broad inhibition of proteasome activity can narrow the therapeutic window and limit dose intensity. Together, these observations suggest that more selective targeting of regulatory proteasome functions may preserve antitumor efficacy while overcoming resistance mechanisms associated with conventional 20S inhibition. Importantly, the limited clinical advancement of several next-generation UPS-targeting agents highlights the challenges of translating promising preclinical findings into effective therapies. In many cases, insufficient selectivity, unfavorable pharmacokinetic properties, and concerns about systemic toxicity have hindered clinical development. These outcomes underscore the need for biomarker-guided patient selection, rational combination therapies, and targets that exploit cancer-specific dependencies rather than globally suppressing proteasome function. Collectively, the successes and shortcomings of earlier proteasome-targeting strategies provide important guidance for the development of future 19S-directed therapeutics.

Despite compelling preclinical activity, no 19S-directed therapy has yet achieved clinical approval. This contrasts sharply with the success of 20S proteasome inhibitors and highlights a major translational gap in the field. A key lesson from previous drug-development efforts is that disruption of essential proteostasis pathways can produce a narrow therapeutic window, necessitating improved target selectivity and more precise patient stratification. Several candidate agents targeting RPN11, RPN13, USP14, and UCHL5 have demonstrated promising antitumor activity in preclinical models, but concerns regarding toxicity, pharmacologic optimization, and target specificity have slowed clinical progression. A summary of clinically relevant proteasome-targeting agents and their associated indications is provided in Table 2.

**TABLE 2:** Clinical trials targeting the ubiquitin-proteome system.

UPS targets	Agent(s)	Disease(s)	Phase	Trial number
20S	Ixazomib	Multiple Myeloma	II	NCT02499081
20S	Ixazomib Citrate and Nelfinavir	Solid Tumors and Lymphoma	I	NCT03422874
20S	Bortezomib	Multiple Myeloma	II	NCT00216697
20S	Bortezomib and Cyclophosphamide	Acute Lymphoblastic Leukemia	III	NCT03646276
20S	Bortezomib and Temozolomide	Solid Tumors and Melanoma	I and II	NCT00512798
AKT	Sorafenib	Uveal Melanoma	II	NCT01377025
ALDH	Disulfiram	Pancreatic Cancer	I	NCT02671890
CRL4CRBN	Lenalidomide	Multiple Myeloma	I and II	NCT06087653
EGFR	Bortezomib Cetuximab and Cisplatin	Head and Neck Cancer	I	NCT01445405

Bortezomib (BTZ) is a reversible proteasome inhibitor that selectively targets the chymotrypsin-like activity of the β5 subunit (PSMB5) within the 20S proteasome, thereby blocking the proteasomal degradation of pro-apoptotic proteins. The resulting accumulation of these factors promotes activation of caspase-mediated apoptosis in malignant cells, which often depend on suppression of apoptotic signaling to sustain proliferation and survival. The clinical success of BTZ has established targeting the UPS as an effective therapeutic strategy in cancer treatment (Kim et al., 2023). Accordingly, BTZ has been approved for the treatment of multiple malignancies, including multiple myeloma and relapsed mantle cell lymphoma, with additional use in other hematologic and select solid tumors. Despite its efficacy, the clinical utility of BTZ is frequently constrained by dose-limiting toxicities, suboptimal pharmacokinetics, and the emergence of drug resistance. Common adverse effects include peripheral neuropathy, thrombocytopenia, gastrointestinal disturbances, fatigue, and, in some cases, cardiac complications, often necessitating dose reduction or treatment discontinuation (Mohan et al., 2017). These limitations highlight the need for next-generation therapeutic strategies that improve tolerability and overcome resistance associated with 20S-directed proteasome inhibition.

Bortezomib (BTZ) resistance arises through multiple mechanisms, including alterations in proteasome subunits, activation of compensatory protein degradation pathways, induction of cellular stress responses, and protective influences from the tumor microenvironment (Patino-Escobar et al., 2023). Among these, mutations in the catalytic proteasome subunits, particularly PSMB5, play a critical role in resistance development, especially in multiple myeloma. Structural mutations within or adjacent to these active-site regions can induce conformational changes that reduce BTZ binding affinity, thereby diminishing proteasome inhibition. In addition, compensatory upregulation of catalytic subunits, particularly PSMB5, can further attenuate drug sensitivity by restoring proteolytic capacity. Collectively, these adaptations enable malignant cells to maintain proteasome function despite inhibitor exposure, ultimately reducing therapeutic efficacy.

To counteract the development of BTZ resistance, combination therapeutic strategies have been actively explored, including the co-administration of bortezomib with menadione sodium bisulfate (MSB). MSB is a synthetic derivative of vitamin K3 that promotes the generation of reactive oxygen species (ROS), thereby increasing oxidative stress within cancer cells. Elevated ROS levels lead to the accumulation of damaged and misfolded proteins, exacerbating proteotoxic stress and enhancing apoptotic signaling pathways. When combined with BTZ, MSB sensitizes malignant cells to proteasome inhibition by overwhelming cellular protein quality-control systems, thereby amplifying the cytotoxic effects of proteasome blockade. This synergistic interaction not only enhances the anti-tumor efficacy of BTZ but also helps circumvent resistance mechanisms driven by proteasome mutations and adaptive cellular responses. Consequently, BTZ–MSB combination therapy represents a promising strategy to reinforce proteotoxic stress and promote apoptosis in resistant cancer cells (Astakhova et al., 2018; Le Guerroué and Youle, 2021; Church and Margolis, 2024).

Hematologic malignancies exhibit some of the most profound dependencies on the ubiquitin–proteasome system, reflecting the exceptional metabolic and proteostatic demands of transformed lymphoid and myeloid cells. While Section 2 outlines the general mechanisms by which proteasome dysregulation contributes to tumorigenesis, a disease-specific perspective is essential for understanding how 19S-directed therapeutic strategies may be translated into clinically meaningful interventions. Unlike many solid tumors, leukemias, lymphomas, and plasma cell malignancies frequently depend on proteasome-regulated pathways such as NF-κB signaling, antigen receptor signaling, protein quality control, and immunoglobulin synthesis. These unique biological features contribute to the remarkable sensitivity of hematologic cancers to proteasome-targeted therapies and provide a strong rationale for the development of next-generation approaches directed at the 19S regulatory particle. The following subsections examine major hematologic malignancies in which 19S proteasome biology has therapeutic relevance, highlighting disease-specific mechanisms of proteasome dependency, emerging vulnerabilities, and opportunities for selective modulation of proteasome function.

### 2.1 Multiple myeloma (MM)

MM remains the hematologic malignancy in which proteasome-targeted therapy has achieved the greatest clinical success. Plasma cells produce large quantities of immunoglobulins, creating a substantial burden on protein quality control pathways and rendering MM cells highly dependent on the UPS. This dependence underlies the clinical efficacy of 20S proteasome inhibitors, including bortezomib, carfilzomib, and ixazomib, although intrinsic and acquired resistance remain major therapeutic challenges (Gandolfi et al., 2017).

Increasing evidence implicates components of the 19S regulatory particle in both proteasome function and therapeutic response (D'Arcy et al., 2015). PSMD14 (RPN11), ADRM1 (RPN13), USP14, and UCHL5 have emerged as promising therapeutic targets whose inhibition disrupts substrate processing and induces proteotoxic stress and apoptosis in preclinical MM models (Tian et al., 2014; Song et al., 2017). Alterations in the 19S ATPases have also been implicated in resistance, including PSMC2 mutations identified in patients with relapsed MM (Haertle et al., 2023). These findings support targeting the 19S proteasome as a complementary strategy to conventional 20S inhibition in proteasome inhibitor-resistant MM.

### 2.2 Diffuse large B-Cell lymphoma (DLBCL)

DLBCL relies on UPS-mediated protein turnover to sustain oncogenic pathways that govern cell survival, proliferation, and stress adaptation, including NF-κB signaling and cell cycle regulation (Bu et al., 2014). Clinical responses to first-generation 20S proteasome inhibitors in DLBCL have been variable, reflecting disease heterogeneity and adaptive resistance mechanisms.

Components of the 19S regulatory particle, particularly ADRM1 (RPN13) and the deubiquitinating enzymes USP14 and UCHL5, have demonstrated antitumor activity in preclinical lymphoma models. Their inhibition disrupts substrate recognition and processing, promotes proteotoxic stress, and activation of apoptotic cell death pathways (Delforoush et al., 2017). Because these targets function upstream of 20S proteolysis, selective 19S-inhibition may provide an alternative approach for overcoming resistance to conventional proteasome inhibitors in DLBCL.

### 2.3 Mantle cell lymphoma (MCL)

MCL exhibits sensitivity to proteasome inhibition, as evidenced by the clinical approval of bortezomib for relapsed or refractory disease (Kane et al., 2007). However, the development of resistance limits durable responses and supports investigation of alternative strategies that exploit proteasome dependence.

The 19S-associated deubiquitinating enzymes USP14 and UCHL5 have emerged as potential therapeutic targets in MCL. Pharmacologic inhibition of these enzymes disrupts substrate processing, promotes proteotoxic stress, and induces apoptotic cell death in preclinical models (Kropp et al., 2018). Although clinical evaluation of 19S-directed therapies in MCL remains limited, these findings support continued investigation of regulatory proteasome components as complementary targets for improving treatment durability.

### 2.4 Acute lymphoblastic leukemia (ALL)

Acute lymphoblastic leukemia (ALL) cells exhibit a substantial dependence on the ubiquitin–proteasome system (UPS) to maintain protein homeostasis, support rapid proliferation, and suppress apoptotic signaling (Houghton et al., 2008; Horton et al., 2019). This reliance on proteostasis mechanisms renders leukemic cells particularly vulnerable to disruptions in proteasome function.

Inhibition of DUBs associated with the 19S regulatory particle, including USP14 and UCHL5, has been shown to impair protein turnover and increase proteotoxic stress in hematologic malignancy models, suggesting potential relevance in ALL as well (Lei et al., 2021). However, compared with other hematologic cancers, the specific contributions of individual 19S regulatory components to ALL pathogenesis, disease maintenance, and therapeutic response remain incompletely defined. Further mechanistic and translational studies are needed to define the roles of individual 19S subunits and associated DUBs in leukemic cell survival and to determine whether selective targeting of these components can enhance therapeutic efficacy, overcome drug resistance, and improve clinical outcomes in ALL.

### 2.5 Chronic lymphocytic leukemia (CLL)

Chronic lymphocytic leukemia (CLL) cells rely on the UPS to regulate signaling networks in survival, proliferation, and resistance to apoptosis including B-cell receptor (BCR) and NF-κB signaling (Pakjoo et al., 2024). Although first-generation proteasome inhibitors have shown limited clinical efficacy as monotherapy, targeting regulatory components of the UPS may provide alternative therapeutic opportunities (Gupta et al., 2013).

In particular, inhibition of the 19S-associated DUBs linked to the 19S regulatory particle has shown antitumor activity in several hematologic malignancies by impairing substrate processing, promoting the accumulation of polyubiquitinated proteins, and inducing proteotoxic stress (Lei et al., 2021). However, the specific functions of individual 19S regulatory components in CLL pathogenesis remain incompletely understood, warranting further investigation to identify therapeutically actionable vulnerabilities.

Collectively, studies across multiple hematologic malignancies highlight the 19S regulatory particle as an emerging therapeutic target with the potential to complement or enhance conventional proteasome inhibition. Figure 3 summarizes the diverse strategies currently under investigation for targeting the 26S proteasome in cancer therapy. Given that acquired resistance remains a major limitation of proteasome-directed treatments, the following section examines the molecular mechanisms underlying resistance to proteasome inhibitors and discusses how targeting the 19S proteasome may provide opportunities to overcome these barriers and improve therapeutic outcomes.

**FIGURE 3:**
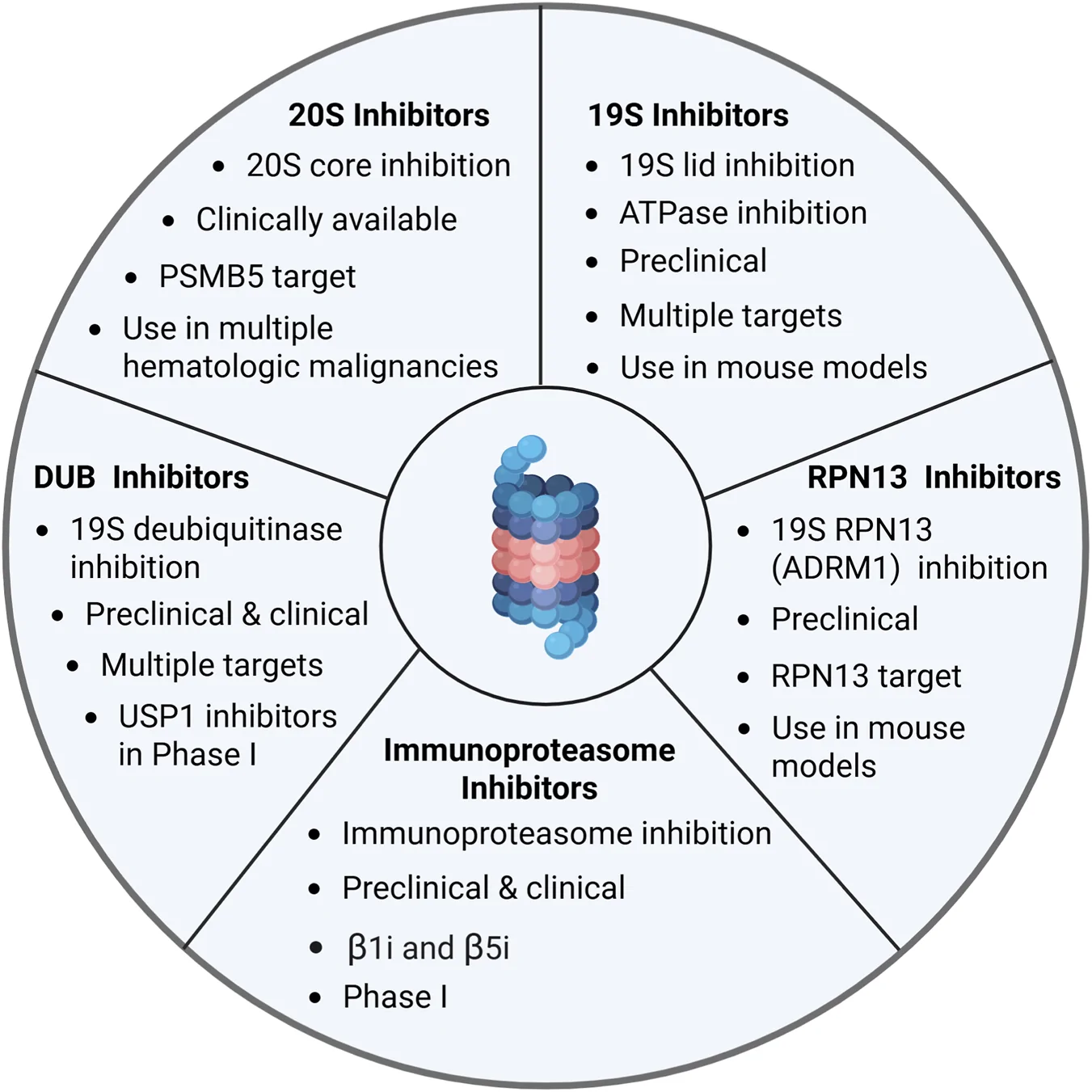
Therapeutic strategies targeting the ubiquitin-proteasome system (UPS). The image summarizes the types of inhibitors used in targeting the UPS, including 20S inhibitors, 19S ATPase inhibitors, deubiquitinase (DUB) inhibitors, RPN13 inhibitors, and immunoproteasome inhibitors. Created in https://BioRender.com.

## 3 Mechanisms of resistance to proteasome inhibitors

Proteasome inhibitors such as bortezomib, carfilzomib, and ixazomib have transformed the therapeutic landscape for several cancers, most notably multiple myeloma and other hematologic malignancies. Although these agents elicit meaningful clinical responses, their long-term efficacy is frequently compromised by both intrinsic and acquired resistance (Patino-Escobar et al., 2023). Tumor cells can adapt to proteasome inhibition through diverse mechanisms, restoring proteostasis, activating compensatory survival pathways, or reducing drug engagement with the proteasome. A comprehensive understanding of these resistance mechanisms is essential for improving the durability of current therapies and for informing the development of next-generation strategies. Accordingly, the following sections highlight the principal molecular and cellular mechanisms that drive resistance to proteasome inhibitors.

### 3.1 Alterations in proteasome subunits

One of the best-characterized mechanisms of resistance to proteasome inhibitors involves alterations in proteasome subunit expression, most notably the β5 subunit encoded by *PSMB5* (Figure 4) (Lu and Wang, 2013). The β5 subunit harbors the chymotrypsin-like proteolytic activity of the 20S proteasome core and is the primary target of clinically used proteasome inhibitors like bortezomib and carfilzomib (Lu and Wang, 2013). Consequently, changes affecting this subunit can directly compromise inhibitor efficacy. Resistance may arise through point mutations in PSMB5 that alter the structural conformation of the β5 active site, reducing inhibitor binding while preserving catalytic activity. In addition, adaptive overexpression of PSMB5 in response to proteasome inhibitor-induced stress enhances chymotrypsin-like activity, thereby partially restoring proteasome function despite pharmacologic inhibition. Together, these mechanisms enable tumor cells to maintain proteolytic capacity and diminish the therapeutic impact of proteasome inhibition.

**FIGURE 4:**
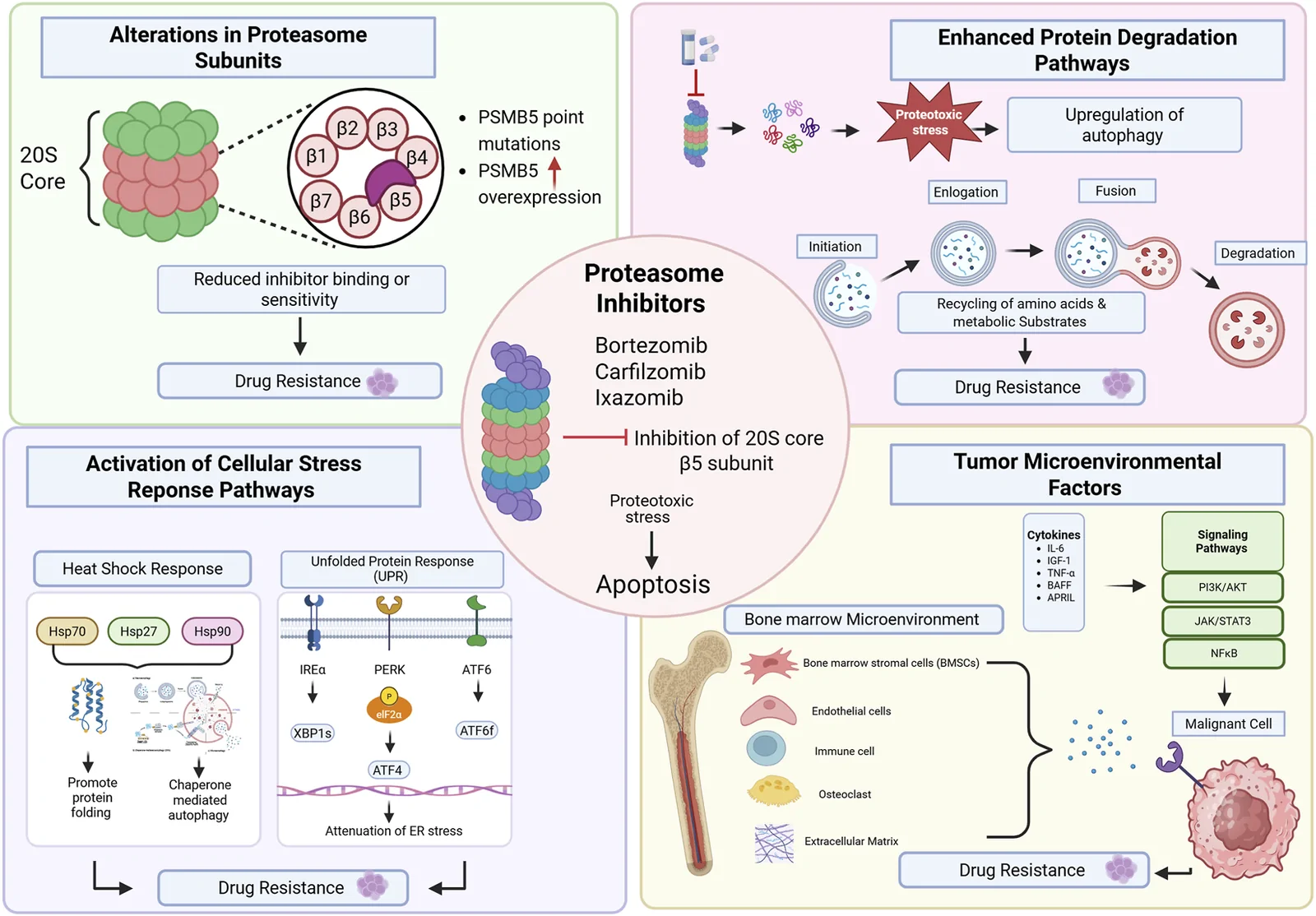
Molecular and cellular mechanisms underlying proteasome inhibitor resistance. The image summarizes the major mechanisms of proteasome inhibitor resistance, including structural alterations in proteasome subunits, most notably PSMB5 point mutations and PSMB5 overexpression-compensatory enhancement of alternative protein degradation pathways such as autophagy, activation of cellular stress response programs involving chaperones (HSP70, HSP27, and HSP90) and the unfolded protein response, and tumor microenvironment-derived influences, particularly signals from the bone marrow niche and the cytokine-mediated modulation of pro-survival signaling pathways. Created in https://BioRender.com.

Importantly, the β5 subunit is not the only proteasome component aberrantly expressed in hematologic malignancies. Multiple independent studies have identified additional 19S regulatory components that contribute to disease progression and therapeutic resistance. For example, the ATPase subunits PSMC2, PSMC3, PSMC4, and PSMC5 are overexpressed in certain acute myeloid leukemia (AML) patients and are associated with inferior overall survival (Tychhon et al., 2023). Likewise, the deubiquitinase RPN11 (PSMD14) and the ubiquitin receptor ADRM1 (RPN13), together with the associated DUB enzymes USP14 and UCHL5, regulate substrate processing and have emerged as promising therapeutic targets in leukemia, lymphoma, and multiple myeloma (Song et al., 2016; Ding et al., 2023; He et al., 2023; Xue et al., 2023). These proteins regulate distinct yet complementary steps of proteasome function, including substrate recognition, deubiquitination, ATP-dependent unfolding, and substrate translocation, highlighting multiple therapeutic vulnerabilities within the 19S regulatory particle. Together, these findings suggest that targeting multiple regulatory functions of the 19S proteasome may provide a more effective strategy for overcoming resistance to proteasome inhibitors than targeting individual subunits alone.

### 3.2 Enhanced protein degradation pathways

In addition to proteasome-centered mechanisms, resistance to proteasome inhibitors can arise through activation of compensatory protein degradation pathways that help preserve cellular proteostasis (Figure 4). Among these, the autophagy–lysosome pathway represents a key adaptive response. Inhibition of proteasomal activity by agents such as bortezomib and carfilzomib leads to the accumulation of misfolded and ubiquitinated proteins, thereby triggering an increase in autophagic flux to alleviate proteotoxic stress (Lu and Wang, 2013). Autophagy facilitates the sequestration of protein aggregates into double-membrane vesicles known as autophagosomes, which subsequently fuse with lysosomes for degradation (Bolandi et al., 2026). This process provides an alternative route for protein clearance when proteasomal function is compromised (Elshazly et al., 2025). Beyond its role in protein quality control, autophagy also supports cellular survival by recycling amino acids and other metabolic substrates under conditions of stress. Importantly, studies in gynecologic and other malignancies have demonstrated that autophagy is upregulated in PI-resistant cells. Moreover, pharmacological or genetic inhibition of autophagy can restore sensitivity to proteasome inhibitors, underscoring its function as a pro-survival mechanism that contributes to therapeutic resistance (Bi et al., 2022).

### 3.3 Activation of cellular stress response pathways

Another important mechanism of resistance to proteasome inhibitors involves activation of cellular stress response pathways, particularly molecular chaperones, which enable cancer cells to adapt to proteotoxic stress induced by proteasome inhibition (Figure 4). Heat shock proteins (HSPs), including HSP70, HSP27, and HSP90, play central roles in maintaining protein homeostasis by promoting proper protein folding, preventing aggregation, and stabilizing damaged or misfolded proteins. Their upregulation in proteasome inhibitor-resistant cells underscores their importance as a protective mechanism that supports cell survival under conditions of proteasome blockade (Shah et al., 2015). Mechanistically, induction of HSPs is often mediated by activation of stress-responsive transcription factors such as heat shock factor 1 (HSF1), which is triggered in response to the accumulation of unfolded proteins. Elevated HSP activity can buffer proteotoxic stress by promoting protein refolding and coordinating with other quality control pathways, including autophagy, thereby diminishing the cytotoxic effects of PIs. These adaptive stress responses enable tumor cells to withstand sustained proteasome inhibition, highlighting molecular chaperones as key contributors to therapeutic resistance and as potential targets for combination strategies aimed at enhancing PI efficacy (Nikesitch et al., 2021).

Importantly, the accumulation of misfolded proteins under conditions of proteasome inhibition imposes substantial stress on the endoplasmic reticulum, leading to activation of the unfolded protein response (UPR) (Kambhampati and Wiita, 2020). The UPR’s primary function is to restore ER proteostasis by transiently attenuating global protein translation, upregulating molecular chaperones, and enhancing the degradation of damaged proteins. The UPR is mediated by three principal ER transmembrane stress sensors: protein kinase RNA-like ER kinase (PERK), activating transcription factor 6 (ATF6), and inositol-requiring enzyme 1 (IRE1), each of which activates distinct but coordinated signaling pathways (Kambhampati and Wiita, 2020). Through these pathways, cells attempt to mitigate proteotoxic stress and maintain protein-folding capacity. However, in the context of proteasome inhibition, sustained UPR activation can promote cell survival by enhancing adaptive stress responses, thereby contributing to resistance to proteasome inhibitors. Under prolonged or overwhelming stress, the UPR can also shift toward pro-apoptotic signaling, highlighting its dual role in determining cell fate (Kambhampati and Wiita, 2020).

Under proteasome inhibition, impaired degradation of ubiquitinated proteins exacerbates proteotoxic stress and drives sustained activation of UPR signaling. While acute UPR activation can trigger apoptosis in response to severe ER stress, chronic, or adaptive signaling instead promotes tumor cell survival and contributes to resistance to proteasome inhibitors. In resistant cells, enhanced UPR activity is associated with increased protein folding capacity, attenuation of ER stress-induced apoptosis, and improved tolerance to proteotoxic burden (Kambhampati and Wiita, 2020). Notably, activation of the PERK/eIF2a and IRE1/XBP1 signaling axes has been implicated in resistance to proteasome inhibitors such as bortezomib and carfilzomib, underscoring the UPR as a central adaptive mechanism in this context (Kambhampati and Wiita, 2020). Collectively, these coordinated signaling pathways enable cancer cells to restore proteostasis under conditions of proteasome inhibition, thereby diminishing the cytotoxic effects of agents such as bortezomib and carfilzomib. These findings underscore the UPR as a key adaptive mechanism driving therapeutic resistance.

### 3.4 Tumor microenvironmental factors

Tumor microenvironment-mediated resistance constitutes a major extrinsic mechanism by which malignant cells evade the cytotoxic effects of proteasome inhibitors, thereby diminishing therapeutic responsiveness (Figure 4). In hematologic malignancies, particularly multiple myeloma and leukemia, tumor cells reside within the bone marrow niche, where they engage in dynamic and reciprocal interactions with bone marrow stromal cells (BMSCs), endothelial cells, osteoclasts, immune cells, and extracellular matrix components (Ria and Vacca, 2020). Mechanistically, cell adhesion-mediated signaling and the secretion of soluble factors, including cytokines, chemokines, and growth factors, activate pro-survival pathways such as NF-κB, PI3K/AKT, and MAPK signaling. These pathways enhance resistance to proteotoxic stress, suppress apoptotic signaling, and protect tumor cells from the effects of proteasome inhibition. Moreover, the bone marrow microenvironment can influence proteasome activity indirectly by modulating oxidative stress, inflammatory signaling, and the expression of stress-response pathways, including molecular chaperones and the UPR. Collectively, these extrinsic protective cues enable tumor cells to buffer the effects of proteasome inhibition and contribute to disease persistence and relapse (Ria and Vacca, 2020).

A central and well-characterized contributor to microenvironment-mediated resistance is cell adhesion–mediated drug resistance (CAM-DR), wherein direct adhesion of malignant cells to stromal components or extracellular matrix proteins activates intracellular signaling networks that suppress apoptosis and promote cell survival (Dalton, 2003). Beyond direct cell–cell and cell–matrix interactions, stromal cells secrete a diverse array of cytokines and growth factors, including interleukin-6 (IL-6), insulin-like growth factor-1 (IGF-1), tumor necrosis factor-α (TNF-α), B-cell activating factor (BAFF), and a proliferation-inducing ligand (April), all of which further reinforce a pro-survival microenvironment (Dalton, 2003). These soluble mediators activate key oncogenic pathways, most notably PI3K/AKT, JAK/STAT, and NF-κB signaling, thereby enhancing resistance to apoptosis and diminishing the cytotoxic effects of proteasome inhibition. Collectively, both adhesive interactions and paracrine signaling within the tumor microenvironment contribute to a robust survival advantage, enabling malignant cells to evade proteasome inhibitor–induced stress and sustain disease progression (Dalton, 2003).

The activation of PI3K/AKT, JAK/STAT3, and NF-κB leads to the upregulation of anti-apoptotic proteins such as BCL-XL, MCL-1, survivin, XIAP, and FLIP (McConkey and Zhu, 2008; Manier et al., 2012). As a result, tumor cells exhibit reduced susceptibility to the proteotoxic stress and apoptotic signaling elicited by proteasome inhibition. Notably, BMSCs can also induce atypical NF-κB activation that persists despite bortezomib treatment, effectively circumventing one of the drug’s primary mechanisms of action (McConkey and Zhu, 2008; Manier et al., 2012).

Collectively, these diverse resistance mechanisms, including structural alterations in 20S catalytic subunits, activation of compensatory degradation pathways such as autophagy, induction of adaptive stress responses including molecular chaperones and the UPR, and protective cues derived from the tumor microenvironment, converge to restore proteostasis and sustain tumor cell survival despite proteasome inhibition. These adaptations underscore a critical limitation of therapies that exclusively target the 20S catalytic core. In contrast, targeting the 19S regulatory particle offers a mechanistically distinct strategy by disrupting upstream processes essential for proteasome function, including substrate recognition, deubiquitination, and ATP-dependent unfolding. By interfering with these rate-limiting steps, 19S-directed approaches may bypass resistance mechanisms driven by catalytic-site mutations, prevent compensatory protein clearance, and overwhelm adaptive stress response pathways. Furthermore, because the 19S complex integrates signals from ubiquitination machinery and cellular stress networks, its inhibition has the potential to disrupt both intrinsic and microenvironment-mediated survival pathways. Together, these features position the 19S proteasome as a promising therapeutic target capable of overcoming the multifactorial resistance associated with conventional 20S-directed proteasome inhibitors (Table 3).

**TABLE 3:** Targeting the 19S proteasome to overcome resistance to 20S inhibition.

Resistance mechanism	Can 19S targeting overcome resistance?	Evidence
PSMB5	Yes	(Oerlemans et al., 2008; Du et al., 2021)
Autophagy	Partial	(Kim et al., 2018)
UPR Activation	Possible	(Zhao et al., 2016; Du et al., 2021)
Bone marrow niche	Possible	(Song et al., 2016)

## 4 The 19S proteasome as a novel UPS target in cancer

Although clinically approved proteasome inhibitors targeting the 20S catalytic core have demonstrated substantial efficacy in multiple myeloma and certain lymphomas, their long-term effectiveness is limited by intrinsic and acquired resistance (Patino-Escobar et al., 2023). In this context, targeting the 19S regulatory particle offers a complementary and mechanistically distinct strategy by disrupting upstream processes essential for proteasome function, including substrate recognition, deubiquitination, and ATP-dependent unfolding. The 19S complex also participates in non-proteolytic functions, including transcriptional regulation, DNA damage responses, and chromatin remodeling, further expanding its therapeutic relevance beyond conventional 20S inhibition (Russell et al., 1999).

Multiple cancers exhibit upregulation of specific 19S subunits, including ATPases (PSMCs) and non-ATPases (PSMDs), which are frequently associated with advanced disease and poor clinical outcomes (Tychhon et al., 2023). Importantly, malignant cells remain highly dependent on 19S-mediated proteasome functions despite resistance to conventional proteasome inhibitors. Targeting 19S components can disrupt these dependencies by inducing the accumulation of polyubiquitinated proteins, triggering apoptosis in a p53-independent manner, and overcoming resistance to agents such as bortezomib and carfilzomib (Du et al., 2021). For example, PSMD1 has been identified as a key therapeutic vulnerability in proteasome-inhibitor-resistant multiple myeloma, with nanoparticle-delivered siRNA causing tumor regression and prolonged survival *in vivo* (Du et al., 2026). Targeting the 19S ATPase Rpt4 has similarly demonstrated selective cytotoxicity in chemotherapy-resistant colon cancer cells while sparing normal tissues (McCawley et al., 2012).

Several pharmacologic strategies have been developed to disrupt 19S regulatory functions. Small molecules such as b-AP15, capzimin, and emerging UCHL5-selective inhibitors target proteasome-associated DUBs, promoting accumulation of polyubiquitinated proteins and proteotoxic stress while retaining activity in models resistant to conventional 20S inhibitors (D'Arcy et al., 2011; Moon et al., 2021; Han et al., 2026). Additional approaches targeting 19S ATPase function or substrate recognition further expand the mechanistic diversity of 19S-directed therapies and may circumvent resistance driven by alterations in 20S catalytic subunits.

USP14, UCHL5, and PSMD14 (RPN11) have emerged as promising therapeutic targets. RPN11 is a metalloprotease DUB that removes polyubiquitin chains immediately before substrate translocation into the 20S core (Verma et al., 2002). Its inhibition disrupts substrate processing, promotes proteotoxic stress, and induces apoptosis. (Liu et al., 2023). The small-molecule inhibitor capzimin selectively targets PSMD14 and has demonstrated potent antitumor activity in preclinical models, including malignancies resistant to conventional proteasome inhibitors (Li et al., 2017), supporting PSMD14/RPN11 as a therapeutic vulnerability within the 19S regulatory particle.

Ubiquitin receptors within the 19S regulatory particle also represent compelling therapeutic targets. RPN13 (ADRM1) functions as a key substrate-recognition receptor that binds polyubiquitinated proteins and facilitates their delivery to the proteasome. Notably, RPN13 is frequently overexpressed across multiple malignancies and has been associated with tumor progression and adverse clinical outcomes. Pharmacologic inhibition of RPN13 using compounds such as RA190 disrupts substrate engagement, induces proteotoxic stress and apoptosis in cancer cells, and has demonstrated activity in bortezomib-resistant multiple myeloma models (Anchoori et al., 2013).

Collectively, genetic and pharmacologic evidence supports the 19S regulatory particle as a distinct therapeutic vulnerability in proteasome inhibitor-resistant cancers. By targeting complementary regulatory functions, including substrate recognition, deubiquitination, and ATP-dependent processing, 19S-directed strategies may bypass resistance associated with conventional 20S inhibition and broaden the therapeutic potential of proteasome-targeted therapies (Table 3).

## 5 Targeting the 19S proteasome in hematologic malignancies

Increasing evidence links aberrant expression of 19S proteasome subunits to adverse clinical outcomes and treatment resistance in hematologic malignancies. PSMD14 is overexpressed in multiple myeloma, where elevated expression correlates with inferior overall survival and reduced response to bortezomib-based therapy. Pharmacological inhibition of PSMD14 restores sensitivity to proteasome inhibitors and exhibits synergistic anti-myeloma activity (He et al., 2023). Similarly, recent studies have identified PSMD1 as a critical therapeutic vulnerability in multiple myeloma, demonstrating that PSMD1 overexpression is associated with poor prognosis and that its depletion can overcome proteasome inhibitor resistance (Du et al., 2026). In AML, elevated expression of several ATPase subunits of the 19S base complex, particularly PSMC2–5, has been associated with unfavorable survival outcomes (Tychhon et al., 2023). Together, these findings suggest that individual 19S components function not only as regulators of proteostasis but also as biomarkers of disease aggressiveness and drivers of therapeutic resistance in hematologic cancers.

Chronic myeloid leukemia (CML) is a hematopoietic stem cell-derived malignancy characterized by the excessive proliferation of immature myeloid cells that accumulate in the peripheral blood and bone marrow (Chereda and Melo, 2015). A hallmark genetic lesion of CML is the Philadelphia (Ph) chromosome, generated by a reciprocal chromosomal translocation that produces the BCR::ABL1 fusion oncoprotein. The resulting constitutively active tyrosine kinase drives aberrant signaling through multiple pathways involved in cell proliferation, survival, and genomic instability, serving as both a diagnostic hallmark and a key biomarker for monitoring disease progression (Chereda and Melo, 2015). In the absence of effective treatment, CML can progress from a relatively chronic phase to an aggressive blast phase, resembling acute leukemia and associated with poor prognosis and high mortality (Chereda and Melo, 2015).

AML is another aggressive hematologic malignancy characterized by the accumulation of immature myeloid cells in the bone marrow and peripheral blood, leading to impaired hematopoiesis and bone marrow failure (Döhner et al., 2022; Lara et al., 2022). Mutations in the FMS-like tyrosine kinase 3 (FLT3) gene are among the most frequently observed genetic abnormalities in AML. Like BCR::ABL1-driven CML, FLT3-mutated AML is characterized by aberrant activation of tyrosine kinase signaling pathways (Chereda and Melo, 2015; Lara et al., 2022). Consequently, tyrosine kinase inhibitors (TKIs), including imatinib for CML and midostaurin, gilteritinib, and quizartinib for FLT3-mutated AML, have become important components of frontline therapeutic regimens (Fava et al., 2016; Lara et al., 2022).

Although TKIs have dramatically improved clinical outcomes in CML and AML, therapeutic resistance remains a major clinical challenge (Apperley, 2007; Fedorov et al., 2023). Resistance may arise through multiple mechanisms, including acquisition of kinase domain mutations that impair drug binding, activation of compensatory signaling pathways, increased genomic instability, metabolic adaptation, and alterations in protein homeostasis networks. As a result, a significant proportion of patients eventually experience disease progression or relapse despite initial responses to therapy (Lara et al., 2022; Bencomo-Alvarez et al., 2021). Growing evidence suggests that the UPS plays a critical role in the development and maintenance of TKI resistance (Li et al., 2024). Leukemic cells exhibit an increased dependence on protein quality-control mechanisms to sustain oncogenic signaling, manage proteotoxic stress, and maintain survival under therapeutic pressure. In particular, several components of the 26S proteasome, including subunits of the 19S regulatory particle, have been implicated in leukemia pathogenesis, disease progression, and treatment resistance. Studies have demonstrated oncogenic functions for 19S subunits such as PSMD1, PSMD3, PSMD14, and multiple PSMC ATPases, linking their expression to adverse clinical outcomes and therapy resistance in both myeloid and lymphoid malignancies. These findings have generated considerable interest in targeting the proteasome as an alternative or complementary therapeutic strategy for myeloid leukemias. Unlike TKIs, which inhibit a single oncogenic kinase, proteasome-directed therapies disrupt multiple cellular processes simultaneously, including protein turnover, transcriptional regulation, stress response pathways, cell cycle progression, and apoptosis. Consequently, targeting the 26S proteasome, particularly the 19S regulatory particle, may represent a promising approach for overcoming TKI resistance and improving outcomes in patients with CML and FLT3-mutated AML.

Disruption of proteasome function, whether through inhibition of the 20S catalytic core or interference with 19S regulatory activities, can exert significant therapeutic effects; however, cancer cells frequently adapt to these perturbations, ultimately leading to treatment resistance. This adaptive capacity highlights the need for therapeutic strategies that selectively disrupt proteasome function without globally abolishing protein turnover, thereby preserving essential cellular processes while targeting tumor-specific dependencies (Tundo et al., 2020). In this context, modulating regulatory components of the proteasome, particularly within the 19S complex, represents a promising approach to achieve more precise and durable antitumor effects.

By impairing the ability of the 19S complex to process and translocate ubiquitinated proteins, overall proteasomal throughput can be selectively reduced without directly inhibiting the catalytic activity of the 20S core. This upstream disruption allows partial preservation of essential proteolytic functions while preferentially targeting the heightened proteasome dependency of malignant cells. Importantly, such an approach may reduce the selective pressures that drive resistance associated with 20S-directed inhibition. In addition, modulation of 19S activity has been associated with beneficial immunomodulatory effects, including enhanced antigen presentation, activation of natural killer cells, and alterations in autophagy pathways, thereby potentially improving antitumor immune responses (Tundo et al., 2020). Collectively, these features highlight 19S-directed targeting as a strategy capable of selectively disrupting tumor-associated proteostasis while mitigating resistance and enhancing immune-mediated therapeutic outcomes.

Among the numerous components of the proteasome, several subunit classes have emerged as promising therapeutic targets. In particular, the 26S proteasome non-ATPase regulatory subunits (PSMD), the 20S catalytic β subunits (PSMB), and the 26S proteasome ATPase subunits (PSMC) represent key targets for modulating proteasome function while minimizing the risk of acquired resistance (Tundo et al., 2020; Kao et al., 2021). Targeting these distinct subunit families enables selective disruption of complementary aspects of proteasome activity, ranging from catalytic proteolysis (PSMB) to substrate recognition, deubiquitination, and ATP-dependent processing (PSMD and PSMC). This multi-level targeting strategy offers the potential to attenuate proteasome function in a more controlled and mechanistically diverse manner, thereby reducing the likelihood of resistance associated with single-site inhibition. Collectively, these findings support the rationale for developing subunit-specific or combinatorial approaches that exploit vulnerabilities within both the catalytic and regulatory components of the proteasome.

When considering potential PSMD targets within the 19S regulatory complex, particular attention should be given to subunits located at the interface between the lid and base (ATPase ring) regions of the 19S structure (Tundo et al., 2020). Targeting subunits at this critical junction preserves the ability of the 19S particle to recognize and bind ubiquitinated substrates while selectively impairing their translocation into the 20S core particle. As a result, ubiquitinated proteins accumulate upstream of the proteolytic chamber, effectively disrupting proteasome-mediated degradation without abolishing essential substrate recognition functions. This selective modulation of proteasome activity provides a strategic advantage, as it allows attenuation of pathological protein turnover while minimizing the global disruption of proteostasis that can lead to toxicity.

Several PSMD subunits, including PSMD1, PSMD4, PSMD8, and PSMD9, have emerged as promising therapeutic targets because of their strategic positions within the lid-base interface of the 19S regulatory particle (Tundo et al., 2020; Bencomo-Alvarez et al., 2021; Lara et al., 2022; Li et al., 2023). Their location enables selective modulation of proteasome function without directly disrupting the catalytic activity of the 20S core. Targeting these regulatory components can impair substrate processing while preserving the structural integrity of the proteasome, potentially reducing the toxicity and resistance associated with conventional catalytic proteasome inhibitors. Consequently, PSMD subunits represent attractive therapeutic targets for selectively modulating proteasome activity. Emerging preclinical evidence demonstrates that inhibition of PSMD-family proteins suppresses tumor growth and enhances proteotoxic stress in multiple malignancies, including AML, CML, breast, ovarian, and pancreatic cancers (Tundo et al., 2020; Li et al., 2023; Nhim et al., 2024). Collectively, these findings highlight the translational potential of PSMD-directed strategies to exploit tumor-specific proteostasis dependencies while minimizing the risk of acquired resistance.

Recent studies further support the specificity and therapeutic relevance of targeting the 19S regulatory particle. Depletion of 19S lid proteins, including PSMD subunits, has been shown to induce monopolar mitotic spindles, a phenotype not observed upon depletion of other proteasome components. The presence of monopolar spindles leads to severe cellular consequences, such as mitotic arrest, cell death, or incorrect chromosome segregation, all of which compromise genomic stability and cellular viability. Importantly, these findings demonstrate that disruptions to distinct proteasome subcomplexes result in unique and discernible cellular phenotypes, underscoring the functional specificity of the 19S subunit. Collectively, this evidence highlights the central role of the 19S regulatory particle in cell cycle progression and further validates its potential as a targeted therapeutic strategy in cancer treatment (Wang et al., 2017).

Lastly, the PSMC subunits represent another promising class of targets for cancer therapy. The PSMC proteins form the ATPase base of the 19S regulatory complex and are among the final components incorporated into the 19S ATPase ring during assembly (Jia et al., 2022). These subunits are essential for proteasome function, as they mediate the recognition, binding, unfolding, and translocation of ubiquitinated substrates into the 20S core through a ring of six AAA-ATPases. Each PSMC subunit plays a crucial role in regulating proteasome-dependent protein degradation and maintaining cellular homeostasis (Kish-Trier and Hill, 2013).

The PSMC family consists of six variants, including PSMC1, PSMC2, PSMC3, PSMC4, PSMC5, and PSMC6, each contributing uniquely to proteasome activity. Emerging evidence supports the relevance of these subunits in cancer progression. In colorectal cancer, PSMC5 overexpression has been associated with poorer patient survival and altered immune cell infiltration, including reduced B-cell and CD8^+^ T-cell levels and increased neutrophil and macrophage infiltration (He et al., 2021). Functional studies further showed that PSMC5 knockdown suppressed colorectal cancer cell proliferation *in vitro*, and mouse models treated with the PSMC5-specific short hairpin RNA exhibited significantly reduced tumor volume and weight compared with controls *in vivo* (Zhang et al., 2026).

Exploring the roles of the PSMD and PSMC proteasome dysfunction may facilitate the development of novel therapeutic strategies for a wide range of diseases. The proteasome indirectly regulates key inflammatory pathways that contribute to the pathogenesis of numerous medical conditions, including cancer, autoimmune diseases, and neurodevelopmental disorders (Goetzke et al., 2021). The structural and functional diversity of proteasome subunits presents unique opportunities for the development of targeted therapeutic and immunotherapeutic approaches. Beyond the canonical 26S proteasome, specialized proteasome variants such as the immunoproteasome have emerged as important regulators of immune function, antigen presentation, and cellular responses to proteotoxic stress. Given its predominant expression in hematopoietic cells and its influence on sensitivity to proteasome inhibitors, the immunoproteasome represents an additional, clinically relevant target in hematologic malignancies. Next, we turn our attention to targeting the immunoproteasome, a specialized proteasome variant induced under inflammatory conditions and critical for antigen processing and immune regulation.

## 6 Targeting the 19S proteasome in combination with the immunoproteasome

The acquired resistance to currently available 20S core particle (CP) proteasome inhibitors has led to the development of novel proteasome-targeted therapeutic strategies. With a better understanding of the mechanisms leading to resistance to conventional proteasome inhibitors, attention has been focused on alternative proteasome targets that may overcome these limitations. Among these, inhibitors targeting the 19S regulatory particle and the immunoproteasome are considered promising approaches because they target distinct sites within the proteasome compared with conventional 20S proteasome inhibitors. Alongside the development of 19S-targeted therapies, increasing evidence has identified the immunoproteasome as another promising proteasome-targeting strategy. Several studies have shown higher expression of the immunoproteasome than constitutive proteasome subunits in B cell malignancies, suggesting an important role for the immunoproteasome in maintaining protein homeostasis in hematologic malignancies. These observations have provided the basis for the development of immunoproteasome-targeted therapies as a potential treatment strategy (Zhang et al., 2020).

As 19S proteasome and immunoproteasome inhibitors act through different mechanisms, they may have complementary effects, and the combination therapy might be beneficial for achieving optimal proteasome inhibition in cancer cells. The immunoproteasome is a specialized proteolytic complex composed of a catalytic 20S CP and associated regulatory units. The 20S core consists of four stacked heptameric rings arranged as α7β7β7α7. The inner β-rings contain the catalytic subunits responsible for protein degradation, while outer α-rings regulate substrate entry and proteolytic activity through interactions with regulatory complexes (Tripathi et al., 2021).

Under inflammatory conditions, particularly following interferon-γ (IFN-γ) stimulation, the constitutive catalytic subunits β1, β2, and β5 are replaced by the inducible subunits β1i, β2i, and β5i, encoded by *PSMB9* (LMP2), *PSMB10* (MECL-1/LMP10), and *PSMB8* (LMP7), respectively (Niewerth et al., 2013; Tripathi et al., 2021; Tubio-Santamaria et al., 2021). These substitutions alter proteolytic cleavage and enhance the generation of peptides optimal for MHC class I presentation.

IFN-γ also induces the proteasome activator PA28, a heptameric complex of PSME1 (PA28a) and PSME2 (PA29b), which binds the 20S core in an ATP-independent manner to facilitate antigen processing. PA28 can associate with the immunoproteasome or standard proteasome cores and form hybrid assemblies with the 19S regulator. Through IRF1- and STAT1-dependent transcription, IFN-γ coordinately upregulates immunoproteasome subunits, PA28, TAP-1, and MHC class I molecules, promoting efficient antigen presentation to CD8^+^ T cells. Hybrid proteasomes containing mixed standard and immunoproteasome subunits further expand function flexibility in immune responses (Niewerth et al., 2013; Tripathi et al., 2021; Tubio-Santamaria et al., 2021).

While the immunoproteasome is best known for its role in MHC class I antigen processing and presentation, it also contributes to protein homeostasis during oxidative stress and inflammation. During inflammatory responses, IFNs stimulate the production of reactive oxygen species (ROS), which in turn promote oxidative protein damage. Dysfunction of the immunoproteasome leads to the accumulation of misfolded and damaged proteins, resulting in protein aggregates. To mitigate the cytotoxic effects of these aggregates, cells generate aggresomal-like induced structures (ALISs), which are transient cytoplasmic inclusions formed in response to cellular stress conditions (Seifert et al., 2010).

The immunoproteasome participates in a wide variety of biological processes, including cancer, autoimmunity, and inflammation (Tripathi et al., 2021). It plays a critical role in T-cell-mediated immunity by influencing the development, activation, and function of both CD4^+^ and CD8^+^ T-cells (Thomas et al., 2023). Consequently, modulation of immunoproteasome activity has emerged as a potential therapeutic strategy to restrain excessive T-cell activation and pathological inflammatory responses (Wang et al., 2020). Notably, studies have shown that immune cells deficient in PSMB8 and other immunoproteasome subunits are unable to produce key inflammatory cytokines, such as IL-23, IFN-γ, IL-2, IL-4, and IL-5, underscoring the essential role of the immunoproteasome in cytokine production and immune regulation (Tripathi et al., 2021). Furthermore, the immunoproteasome is involved in the activation of Th1 and Th17 responses. Inhibition of specific immunoproteasome subunits, such as LMP7 (β5i), in murine models reduces pro-inflammatory Th1 and Th17 cells while promoting the differentiation of regulatory T cells (Tregs). Collectively, these findings suggest the targeting of specific immunoproteasome subunits as a promising therapeutic approach for the treatment of autoimmune diseases (Kalim et al., 2012).

The immunoproteasome is predominantly expressed in hematopoietic immune cells, including B cells, macrophages, and dendritic cells, and represents the major proteasome form in tumor cells derived from hematological malignancies (Tubio-Santamaria et al., 2021). Importantly, the ratio of immunoproteasome to constitutive proteasome varies across different hematopoietic cancers and has a significant impact on therapeutic response and clinical outcomes. For example, pre-B ALL shows higher immunoproteasome expression, whereas AML exhibits a lower immunoproteasome-to-constitutive proteasome ratio, greater abundance of the standard proteasome. This ratio is a critical determinant of cancer cell sensitivity to proteasome inhibitors (Niewerth et al., 2013; Niewerth et al., 2016).

Although immunoproteasome inhibitors were developed primarily for autoimmune diseases, interest has expanded into cancer therapy. Tumor cells with higher immunoproteasome expression show increased responsiveness to proteasome inhibitor-based therapies. Notably, ALL cells are approximately 5.5-fold more sensitive to proteasome inhibitors than AML cells, which display reduced immunoproteasome levels and greater treatment resistance. These observations suggest that the immunoproteasome/constitutive proteasome ratio may serve as an innovative biomarker for predicting therapeutic efficacy. Furthermore, modulation of this ratio could enhance the efficacy of proteasome inhibitors such as bortezomib, carfilzomib, and ONX-0194 (a selective and irreversible β5i inhibitor) (Niewerth et al., 2013; Sin and Man, 2021). Immunoproteasome expression has also been documented in myeloproliferative neoplasms, further supporting its relevance in hematologic cancers (Niewerth et al., 2013).

Proteasome inhibitors exert their antitumor effects through multiple mechanisms, including suppression of NF-κB signaling by preventing the proteasomal degradation of its inhibitor, IκB. NF-κB is a central transcription factor that promotes tumor cell survival, angiogenesis, and metastasis through the regulation of various genes such as VEGF, BCL2, STAT3, IL-8, TGF-β, and TNF-α (Mohan et al., 2017). In AML, elevated B1i/B1 and B2i/B2 subunit ratios are associated with decreased sensitivity to bortezomib. However, all immunoproteasome-to-constitutive subunit ratios correlate with responsiveness to carfilzomib, which targets both proteasome forms. This finding suggests that increased immunoproteasome levels enhance the sensitivity of AML cells to carfilzomib treatment.

In contrast, ALL cells show no significant correlation between the immunoproteasome/constitutive proteasome ratio and their response to either bortezomib or carfilzomib, likely due to their intrinsically high sensitivity to these inhibitors. However, in ALL, the β1i/β1 subunit ratio correlates with responsiveness to ONX0914, indicating that higher β1i expression is associated with increased susceptibility to selective immunoproteasome inhibition (Jenkins et al., 2021). Collectively, these findings support the use of the immunoproteasome-to-constitutive proteasome ratio as a predictive biomarker that may guide the selection of the most effective and personalized therapeutic strategies in hematologic malignancies.

Targeting the immunoproteasome may complement 19S proteasome inhibition by simultaneously disrupting proteostasis and modulating antitumor immunity. Whereas 19S inhibition induces the accumulation of ubiquitinated proteins and proteotoxic stress through impaired substrate recognition, deubiquitination, and unfolding, immunoproteasome-directed strategies influence antigen processing, inflammatory signaling, and immune surveillance. Combined targeting could prevent adaptive proteasome reprogramming, enhance tumor immunogenicity, and sensitize malignant cells to both proteotoxic stress and immune-mediated elimination, providing a rationale for integrating 19S proteasome inhibitors with immunoproteasome modulators and immunotherapies in hematologic malignancies. Next we discuss the current challenges and limitations of targeting the 19S proteasome in hematologic malignancies.

## 7 Current challenges and limitations of targeting the 19S proteasome

Despite growing evidence that components of the 19S regulatory particle represent promising therapeutic targets in cancer, several challenges have hindered their clinical translation. These include concerns regarding toxicity and target selectivity, the absence of clinically approved 19S-directed agents, activation of compensatory proteostasis pathways, adverse effects on normal hematopoietic cells, and broader pharmacologic obstacles associated with drug development. Addressing these limitations will be essential for realizing the therapeutic potential of 19S-targeted strategies.

### 7.1 Toxicity and essential cellular functions

The 19S regulatory particle plays a central role in substrate recognition, deubiquitination, unfolding, and translocation of ubiquitinated proteins into the 20S catalytic core. As a result, it is indispensable for the maintenance of cellular protein homeostasis in virtually all eukaryotic cells. Unlike many oncogenic signaling pathways that exhibit tumor-specific dependencies, most 19S subunits perform fundamental housekeeping functions required for normal cell survival. Consequently, broad inhibition of 19S activity may induce excessive proteotoxic stress, ER stress, and apoptosis in healthy tissues, potentially leading to significant systemic toxicities (Tsvetkov et al., 2017). These concerns are particularly relevant in highly proliferative tissues such as the bone marrow, gastrointestinal epithelium, and immune system, where disruption of proteostasis can impair normal tissue function and regeneration (Shah et al., 2024; Yu et al., 2025).

### 7.2 Limited selectivity for malignant cells

Achieving a sufficient therapeutic window remains one of the greatest challenges in targeting the 19S proteasome. Cancer cells frequently exhibit increased dependence on proteasome function due to elevated protein synthesis, genomic instability, oxidative stress, and the accumulation of misfolded proteins. However, normal cells also rely heavily on 19S-mediated protein degradation to maintain proteostasis. Many proposed 19S targets, including PSMD14, PSMC family ATPases, and ubiquitin receptors such as ADRM1, are ubiquitously expressed and essential for normal cellular function. Consequently, distinguishing tumor-specific proteasome dependencies from essential housekeeping activities will be critical for developing therapies that maximize antitumor efficacy while minimizing off-target toxicity.

### 7.3 Lack of clinically approved 19S inhibitors

In contrast to clinically approved 20S proteasome inhibitors such as bortezomib, carfilzomib, and ixazomib, no agents that specifically target the 19S regulatory particle have yet entered routine clinical practice. Several compounds directed against PSMD14/RPN11, RPN13, and other 19S-associated proteins have demonstrated significant antitumor activity in preclinical models (Song et al., 2016; Li et al., 2017; Spataro and Buetti-Dinh, 2022). Notable examples include the PSMD14 inhibitor capzimin and several RPN13-targeted agents that induce proteotoxic stress and apoptosis in cancer cells. Nevertheless, these compounds remain largely in preclinical development, and substantial efforts in optimization, toxicity assessment, pharmacokinetic evaluation, and clinical validation are still required before they can be translated into patient care.

### 7.4 Compensatory proteostasis and resistance mechanisms

Cancer cells possess remarkable adaptive capacity and can activate alternative protein quality-control pathways in response to proteasome inhibition. Enhanced autophagy, lysosomal degradation, unfolded protein response (UPR) signaling, and stress-responsive transcriptional programs may partially compensate for impaired 19S function and help restore proteostasis. Furthermore, tumor cells may alter the expression or activity of proteasome subunits, ubiquitin ligases, molecular chaperones, or deubiquitinating enzymes to circumvent proteasome inhibition. These adaptive responses can diminish therapeutic efficacy and contribute to both intrinsic and acquired drug resistance, highlighting the need for combination strategies that simultaneously target complementary proteostasis pathways.

### 7.5 Effects on normal hematopoietic cells

The potential impact of 19S inhibition on normal hematopoiesis represents a particularly important concern in hematologic malignancies. Hematopoietic stem and progenitor cells rely on tightly regulated protein turnover to maintain self-renewal, differentiation, and cellular homeostasis. Disruption of 19S proteasome function may therefore impair normal hematopoietic processes, potentially resulting in cytopenias, immunosuppression, and reduced bone marrow regenerative capacity. These risks are especially relevant in leukemias, where therapeutic strategies must selectively eliminate malignant clones while preserving normal stem cell populations. A deeper understanding of proteasome dependencies in leukemic versus normal hematopoietic cells will be essential for identifying therapeutic windows that allow selective targeting of malignant cells.

### 7.6 Pharmacologic and drug development challenges

Several pharmacological barriers have also limited the development of 19S-directed therapies. Many 19S subunits function within large, dynamic multiprotein complexes characterized by extensive protein-protein interaction surfaces that are inherently challenging to target with conventional small molecules. In addition, ATPase and scaffolding subunits often lack clearly defined druggable pockets, complicating rational drug design. Successful therapeutic candidates must not only engage their intended targets with high specificity but also achieve adequate intracellular exposure and exhibit favorable pharmacokinetic and pharmacodynamic properties. Further complicating clinical development is the lack of robust biomarkers capable of measuring target engagement, identifying responsive tumors, and guiding patient selection.

Overcoming these challenges will likely require a combination of highly selective inhibitors, targeted protein degradation technologies, synthetic lethality-based approaches, and rational combination therapies designed to suppress compensatory survival mechanisms. Advances in structural biology, chemical biology, and proteasome research are beginning to reveal tumor-specific dependencies that may enable more precise therapeutic targeting. As our understanding of 19S proteasome biology continues to expand, particularly in hematologic malignancies, new opportunities are likely to emerge for exploiting regulatory particle vulnerabilities while minimizing toxicity to normal tissues.

## 8 Ongoing drug development and translational challenges for targeting the 19S proteasome

Although numerous studies have validated components of the 19S regulatory particle as promising therapeutic targets in cancer, the development of clinically viable inhibitors remains in its infancy. Significant progress has been made in identifying compounds that selectively disrupt key functions of the 19S proteasome, particularly DUBs, ubiquitin receptors, and ATPase subunits. However, substantial challenges related to target selectivity, toxicity, druggability, biomarker development, and resistance continue to impede clinical translation. Overcoming these barriers will be critical for realizing the therapeutic potential of 19S-directed strategies.

### 8.1 Current status of drug development

Efforts to therapeutically target the 19S proteasome have focused primarily on the deubiquitinating enzyme PSMD14 (Rpn11), which is responsible for removing ubiquitin chains from substrates before their translocation into the 20S catalytic core (Li et al., 2017). Several experimental compounds, including capzimin and related derivatives, have demonstrated potent inhibition of PSMD14 activity, resulting in the accumulation of polyubiquitinated proteins, induction of proteotoxic stress, and suppression of tumor growth in diverse preclinical models. In parallel, agents targeting other components of the 19S regulatory particle, including ubiquitin receptors and ATPase subunits, have shown encouraging antitumor activity in both hematologic and solid malignancies.

Beyond conventional small-molecule inhibitors, emerging therapeutic modalities are expanding the range of strategies available for manipulating proteasome biology. Proteolysis-targeting chimeras (PROTACs), molecular glues, and other targeted protein degradation technologies provide opportunities to selectively modulate proteins within the ubiquitin–proteasome system, extending therapeutic intervention beyond simple catalytic inhibition (Békés et al., 2022; Zhao et al., 2022; Sasso et al., 2023; Hinterndorfer et al., 2025). Concurrently, advances in cryo-electron microscopy have defined the architecture and conformational dynamics of the 26S proteasome at near-atomic resolution, providing an unprecedented structural framework for the rational design of next-generation agents directed against the 19S regulatory particle (Chen et al., 2016; Dong et al., 2019; Arkinson et al., 2025).

### 8.2 Barriers to clinical translation

Despite promising preclinical results, no therapy specifically targeting the 19S proteasome has yet advanced successfully to late-stage clinical development. A major obstacle is the essential role of the 19S regulatory particle in maintaining protein homeostasis in normal tissues. Because many 19S subunits perform indispensable cellular functions, systemic inhibition carries the risk of substantial toxicity and may limit the therapeutic window available for clinical intervention.

A second challenge is the absence of reliable biomarkers capable of identifying tumors that are most likely to respond to 19S-directed therapies. Although many cancers exhibit increased dependence on proteasome activity due to elevated protein synthesis, chromosomal instability, metabolic stress, and the accumulation of misfolded proteins, quantitative measures of proteasome dependency remain poorly established. The lack of validated predictive biomarkers complicates patient selection and may hinder the design of future clinical trials.

Furthermore, cancer cells can activate compensatory proteostasis pathways in response to proteasome inhibition. Enhanced autophagy, lysosomal degradation, unfolded protein response signaling, and stress-adaptive transcriptional programs may partially restore protein homeostasis and reduce therapeutic efficacy. These adaptive mechanisms suggest that 19S-targeted agents may ultimately prove most effective when incorporated into rational combination strategies that simultaneously suppress complementary protein quality-control pathways.

### 8.3 Medicinal chemistry challenges

The structural and functional characteristics of the 19S proteasome present unique challenges for drug discovery (Arkinson et al., 2025). Unlike the catalytic active sites of the 20S core particle, many 19S subunits exert their functions through protein-protein interactions, ATP-dependent conformational remodeling, and dynamic assembly processes (Pei et al., 2026). These features often lack deep, well-defined binding pockets, making them difficult targets for traditional small-molecule approaches.

Moreover, many currently available inhibitors exhibit limitations, including insufficient target selectivity, poor metabolic stability, limited bioavailability, suboptimal pharmacokinetic properties, and unintended activity against related DUBs or ATPases. The large size and conformational flexibility of the 19S complex further complicate structure-based drug design. Because multiple regulatory subunits function within highly coordinated molecular networks, selective modulation of a single component without disrupting essential cellular processes remains a significant challenge.

Nevertheless, recent advances in fragment-based drug discovery, covalent ligand screening, structure-guided drug design, and artificial intelligence-assisted molecular modeling are creating new opportunities to identify ligands capable of engaging previously intractable targets. Continued integration of these technologies with emerging structural and mechanistic insights into proteasome biology may accelerate the development of clinically actionable 19S-directed therapeutics.

## 9 Concluding remarks and future perspectives

Accumulating evidence underscores the central role of the UPS in cancer progression, therapeutic resistance, and cellular adaptation to stress, positioning the 19S regulatory particle as a compelling and relatively underexplored target for next-generation anticancer therapies. Although first-generation 20S proteasome inhibitors have transformed the treatment of multiple myeloma and other hematologic malignancies, their long-term clinical utility is frequently limited by toxicity, incomplete responses, and the development of acquired resistance. Targeting regulatory components of the proteasome, particularly the 19S subcomplex, offers a mechanistically distinct strategy for disrupting oncogenic protein turnover, stress-response pathways, ubiquitin signaling, and proteasome-dependent survival networks without directly inhibiting the catalytic core.

Growing evidence from hematologic and solid tumor models demonstrates that inhibition of specific 19S-associated proteins, including DUB enzymes, ubiquitin receptors, and ATPase subunits, can induce proteotoxic stress, disrupt protein homeostasis, and suppress tumor growth. In parallel, increasing interest in selectively targeting proteasome subtypes, such as the immunoproteasome, further highlights the potential of precision approaches that exploit disease-specific proteasome dependencies while reducing off-target effects. Collectively, these findings support a broader shift toward selective modulation of the UPS as a therapeutic strategy, with the 19S regulatory particle emerging as a promising target for overcoming resistance and expanding the clinical utility of proteasome-directed therapies.

Future success will depend on the development of highly selective agents capable of exploiting tumor-specific proteostasis vulnerabilities while preserving normal cellular function. Rational combination approaches involving autophagy inhibitors, immunotherapies, kinase inhibitors, targeted protein degraders, or conventional proteasome inhibitors may further enhance therapeutic efficacy and limit resistance. Equally important will be the identification of predictive biomarkers, synthetic lethal interactions, and lineage-specific dependencies that enable effective patient stratification and precision treatment strategies. Continued advances in structural biology, medicinal chemistry, cryo-electron microscopy, and targeted protein degradation technologies are likely to accelerate the development of clinically actionable 19S-directed therapeutics. As our understanding of proteasome regulation continues to expand, selective targeting of the 19S regulatory particle may ultimately provide a new generation of therapies capable of overcoming the limitations of current proteasome inhibitors and improving outcomes across a broad spectrum of malignancies.
